# Effects of *Withania somnifera* Extract in Chronically Stressed Adults: A Randomized Controlled Trial

**DOI:** 10.3390/nu16091293

**Published:** 2024-04-26

**Authors:** Srikanta Pandit, Amit K. Srivastav, Tapas K. Sur, Supriyo Chaudhuri, Yan Wang, Tuhin K. Biswas

**Affiliations:** 1 Research Unit, Department of Health & Family Welfare, Government of Bengal, J. B. Roy State Ayurvedic Medical College and Hospital, 170−172, Raja Dinendra Street, Kolkata 700004, India; srikantapandit@gmail.com (S.P.); dramitshrivastava1981@gmail.com (A.K.S.); drtapaskumarsur@gmail.com (T.K.S.); chaudhuridrsupriyo@gmail.com (S.C.); 2 Section of Public and Population Health, School of Dentistry, University of California, Los Angeles (UCLA), Los Angeles, CA 90095, USA; wangyan@ucla.edu

**Keywords:** *Withania somnifera*, ashwagandha, perceived stress scale (PSS)

## Abstract

Background: Stress is a known causative factor in modulating cognitive health, which overall well-being and quality of life are dependent on. Long-term stress has been shown to disrupt the balance of the hypothalamic–pituitary–adrenal (HPA) axis. Adaptogens, such as *Withania somnifera* (ashwagandha), are commonly used in Ayurvedic medicine for stress relief and ameliorating HPA-axis dysfunction. The aim of this study was to support the role of a root and leaf water-extracted ashwagandha extract (WS) in stress reduction by confirming the lowest clinically validated dose for stress management (125 mg/day) in a dose-dependent clinical study in adults with self-reported high stress. Methods: An 8-week, randomized, double-blinded, placebo-controlled study to compare the effects of three different WS extract doses (125, 250 and 500 mg) was performed. A total of 131 adults were enrolled, and 98 were included in the final analysis. Attenuation of chronic stress was measured using the 14-item Perceived Stress Scale (PSS) and biochemical-related stress parameters. Results: We have shown that aqueous WS extract (roots and leaves) safely reduces mild to moderate chronic stress at doses of 125 mg, 250 mg, and 500 mg/day for 8 weeks. Conclusions: Our findings demonstrate the stress-reduction capabilities of this well-characterized aqueous extract of WS (root and leaf) at the low dose of 125 mg/day, in a dose-dependent manner, via the modulation of the HPA axis. Trial registration: This study was registered with the Clinical Trials Registry—India (CTRI) with the registration number: CTRI/2019/11/022100.

## 1. Introduction

Psychological stress plays a significant role in our society, with worldwide prevalence exponentially increasing in recent years [1]. Upon encountering a stressor, a complex cascade of physiological responses is initiated, resulting in the activation of the hypothalamic–pituitary–adrenal (HPA) axis [2] and the secretion of the primary stress hormone cortisol [3]. However, chronic stress or ongoing exposure to stressors can disrupt the balance of the HPA axis, resulting in a prolonged elevation of cortisol levels. This dysregulation may lead to adverse health effects, including anxiety, depression, metabolic disorders, immune dysfunction, cardiovascular disease, and sleep difficulties [4,5,6]. Therefore, strategies for minimizing stress impact and ameliorating stress-related symptoms are of great interest to the general population.

Ashwagandha (*Withania somnifera*), also known as Indian Ginseng or Winter Cherry, is a member of the family of herbs referred to as “adaptogens”, that is, substances that regulate metabolism when a body is perturbed by physical or mental stress and help the body to adapt to this stress [7]. Therefore, this botanical is widely used in Ayurveda, an ancient traditional Indian system of medicine, and has been suggested to promote stress amelioration, restore homeostasis, and exert neuroprotective and immunomodulatory properties [7,8,9,10,11,12]. More recently, clinical studies in humans showed that WS extract is well-tolerated and may reduce stress, anxiety, and depression in stressed subjects [13], exerting its effect by modulating cortisol [14] and testosterone levels [15].

However, existing trials were limited by their small sample sizes and the variety of outcomes used to measure efficacy. Moreover, in these studies, divergent WS extracts with varying treatment doses were used [16]. Therefore, more scientific evidence is needed to corroborate the anti-stress properties of ashwagandha using well-characterized and standardized extracts at defined dosages.

The main aim of this study was to build and support the anti-stress effects and safety profile of a standardized aqueous WS extract of root and leaf (Sensoril^®^, Kolkata, India) in chronically stressed subjects by confirming the lowest clinically validated dose for stress management (125 mg per day) in a dose-dependent clinical study. The secondary aims were to evaluate the effectiveness of this extract in improving sleep and quality of life in this population.

## 2. Materials and Methods

### 2.1. Study Design

This was an 8-week, randomized, double-blind, placebo-controlled study with a parallel-group design; the aim was to compare the effects of three different doses (125 mg, 250 mg, and 500 mg per day) of a WS root and leaf extract (Sensoril^®^). It assessed the effect on the mood, behavioral, and biochemical indices of chronic stress and was conducted in community-dwelling adults who sought help for psychological and other symptoms related to chronic stress. The trial was conducted following the guidelines of the Indian Council of Medical Research (ICMR). The approval number of the Ethical Permission was obtained on 22 August 2019 (JBR/IEC/10; dated 22 August 2019), and the clinical study was retrospectively registered with the Clinical Trials Registry—India (CTRI/2019/11/022100; date of registration 21 November 2019), with participant recruitment occurring between January 2021 and July 2022.

The study was conducted in the Research Unit, Department of Kayachikitsha, J.B. Roy State Ayurvedic Medical College and Hospital, Kolkata, India. Clinical oversight of the study was provided by the Psychopharmacology Unit, Department of Clinical Psychopharmacology and Neurotoxicology, National Institute of Mental Health and Neurosciences, Bangalore, India. We estimated that a sample size of 30 subjects per group (vs. control) would be adequate to detect a moderate effect size of 0.75 for an alpha of 5% and a power of 80% (total *n* = 120). The effect size was based on previous experience with clinical trials of this nature in the J.B. State Ayurvedic Medical College and Hospital. Additional subjects were eligible to be registered considering the drop-out rate.

Potential participants were screened after being informed and providing informed consent, and 131 eligible subjects (40 women and 71 men; mean age 35 years old) were enrolled in the study as per inclusion and exclusion criteria. Volunteers were randomized into blocks of eight to receive either Sensoril^®^ (Prepared by Natreon India, Rishi Tech Park, Premises No.: 02−360, Action Area 1D, New Town, Kolkata, 700 156) or a matching placebo for 8 weeks. There were four groups: (1) subjects who consumed 125 mg/day of the WS extract (WS125 group); (2) subjects who consumed 250 mg/day of the WS extract (WS250 group); (3) subjects who consumed 500 mg/day of the WS extract (WS500 group); and (4) subjects who consumed a placebo (control group). Treatments were given to the subjects for 8 weeks. In all cases, the study products were dispensed in the form of a single capsule at night, approximately half an hour before bedtime. The capsules were formulated by Natreon India and were identical in appearance across the four groups. Neither the researchers nor the subjects knew which treatment sequence the subjects had been assigned to; the researchers were unblinded only at the end of the study.

### 2.2. Study Participants

Study participants met the following inclusion criteria: men and women from 18 to 60 years old suffering from anxiety, depression, and/or sleep disturbances related to chronic stress for >3 months; a total score ≥28 on the Perceived Stress Scale (PSS); and a score ≥3 for Item 3 of the PSS questionnaire. Participants were ineligible for participation in the study if they were pregnant, lactating, intending to become pregnant, or not using any method of birth control. Other exclusion criteria included the presence of untreated or unstable major comorbidities such as diabetes, hypertension, or ischemic heart disease; current or past history of any major medical or neuropsychiatric disorder; the presence of clinically significant medical or psychiatric symptoms for which the initiation of medication was indicated; alcohol or other substance-use disorder; use of any psychotropic or nutraceutical medication for >1 week during the month before study initiation, use of any psychotropic or nutraceuticals drug during the week before the study initiation, or current treatment with psychotherapy; suicidal intent at any time during the four weeks before the intervention; and anticipation of any change in stressors or life events (increase or decrease) across the course of the study.

All subjects invited to participate were informed about the study and its procedures and then signed a written informed consent form.

### 2.3. Study Products

The WS extract used in this study (Trade name Sensoril^®^, Kerry Group, Tralee, Ireland) was produced from ashwagandha cultivated in the central and northern provinces of India; it consisted of leaf and root material processed using a water-based extraction protocol. Full chemical characterization of the ingredient was undertaken, identifying 50 small molecules, including 26 with anolides isolated by multi-step chromatography (MPLC, subsequent HPLCs with different stationary and mobile phases) into pure compounds and further characterized into isolated compounds by LCMS and 1D- and 2D-NMR spectroscopy. Using a validated analytical method that accounts for mid-polar compounds, ten major secondary metabolites (nine withanolides and one flavonoid glycoside) were selected for quantification by UPLC-PDA and were in the range of 0.4–1%.

### 2.4. Study Outcomes and Data Collection

The primary outcome of this study was to determine whether the WS extract attenuates chronic stress using the 14-item Perceived Stress Scale (PSS) [17] and measuring the following biochemical-related stress parameters: salivary alpha-amylase, plasma cortisol, adrenocorticotropin (ACTH), and sulfate adrenal androgen dehydroepiandrosterone (DHEA-S). Secondary outcomes included the attenuation of anxiety and depression, measured by the Hamilton Anxiety Scale (HAM-A) and Hamilton Depression Scale (HAM-D); sleep parameters and vitality improvement, assessed by the Pittsburgh Sleep Quality Index (PSQI), Visual Analogue Scale for Sleep (VAS-S), and Visual Analogue Scale for energy, vitality, and drive (VAS-E); quality of life, using the World Health Organization Quality of Life: Brief Version (WHOQoL-Bref) questionnaire; and the inflammatory parameters hsC-reactive protein, IL−1β, IL−6, and TNF-α. Finally, for the safety assessment, several biochemical parameters were assessed during the study: hemogram; fasting glucose and lipids parameters; and thyroid, liver, and renal function parameters. Also, all volunteers went through a general and systemic physical examination, including body weight measurements.

Enrolled subjects attended the study center five times. The baseline visit took place 2 to 7 days after the screening visit, where demographic and clinical data were collected. Participants were randomized and provided with 1 container of 35 capsules. At visits 2 (2 weeks ± 3 days), 3 (4 weeks ± 5 days), and 4 (end of the intervention; 8 weeks ± 7 days), participants returned to the center for clinical efficacy and safety and tolerability assessments. A count of the returned pills and provision of a new container of pills were performed at visit 3 (4 weeks ± 5 days), and a final end-of-treatment count was performed at the end of the intervention (visit 4; 8 weeks ± 7 days). At visit 1 (baseline) and visit 4 (8 weeks ± 7 days), a morning blood sample was also collected to assess biochemical parameters. There was an additional follow-up at 9 weeks (± 2 days) to assess treatment discontinuation symptoms, if any, and to assign the patient to treatment as usual; this was assessed by the Clinical Institute Withdrawal Assessment Scale—Benzodiazepines (CIWA-B). Adverse events, defined as any unfavorable, unintended effects, were recorded at every follow-up visit (2, 4 and 8 weeks). No concurrent psychotropic or nutraceutical medications were permitted during the study.

### 2.5. Biochemical Parameter Analysis

Salivary alpha-amylase was measured by an ELISA technique (Novas Biologicals, LLC, CO, USA), whereas plasma cortisol, ACTH, and DHEA-S levels were determined using CLIA (Chemiluminescent Immunoassay) instruments (ADVIA Centaur XPT, Siemens Healthineers, Malvern, PA, USA). Regarding inflammatory parameters, hsC-reactive protein was analyzed by a CLIA method (Siemens Healthineers, USA), and plasma levels of IL−1β, IL−6, and TNF-α were analyzed by ELISA Kits (RayBiotech, Norcross, GA, USA). Safety assessment parameters were analyzed by standardized colorimetric methods using an automated blood analyzer by Beckman Coulter CA, USA, at the Serum Analysis Centre, Kolkata, India. However, the thyroid function test panel was measured using a CLIA method (Siemens Healthineers, USA). For a full list, see Table below and the Supplementary Materials.

### 2.6. Statistical Analysis

The normality of the distribution for all measured variables was tested by normal probability plots and the Shapiro–Wilk test. Data are presented as the mean ± standard deviation (SD) for continuous variables and as *n* (%) for categorical variables.

Baseline characteristics were compared between treatment and control groups using the chi-square test for categorical variables and one-way ANOVA for continuous variables.

All efficacy outcomes were compared at baseline and subsequent time points using univariate one-way ANOVA followed by a post-hoc Tukey HSD test to perform pairwise comparisons while controlling by a family-wise error rate.

To further control the type I error rate and derive summary estimates of group differences throughout the entire intervention period, two longitudinal mixed models (LMMs) were used to model the continuous efficacy outcomes. These models allowed us to adjust for potential confounders and account for individual intervariability. The covariance structure was modeled using first-order autoregression, i.e., the current observation is correlated with the last observation.

All models were adjusted for sex, age, BMI, and the corresponding baseline measure. The model can be written as:

Continuous scale = β0 + β1∗[week] + β2∗[group] +β3∗[week∗group] + β4∗[covariates] + error

Model 1 compared all treatment groups versus the placebo group, while Model 2 compared lower (125 mg) and higher dosage levels (500 mg) versus the middle dosage level (250 mg).

All analyses were conducted using SAS 9.4. The significance level was set at 0.05. We did not adjust for the false discovery rate, as the main purpose of the study was not to identify biomarkers but to compare the treatment groups versus the placebo group and indicate the effectiveness of the dosage levels.

## 3. Results

### 3.1. Study Data, Compliance, and Baseline Characteristics of the Subjects

**Figure 1:**
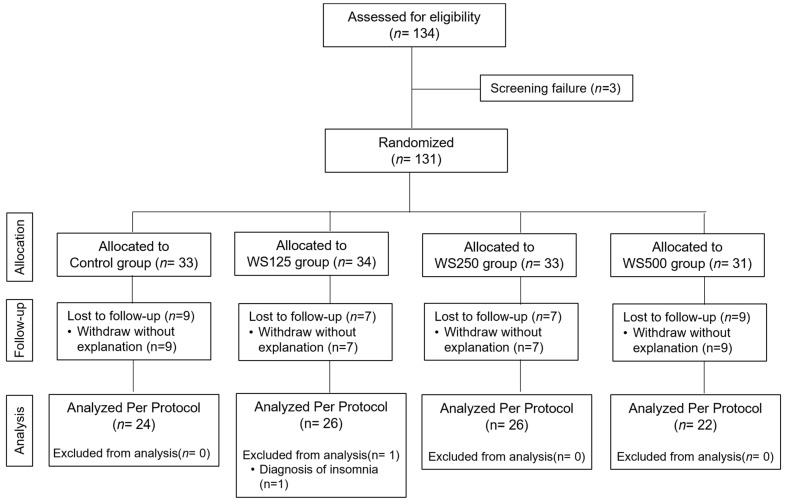
Participant disposition (CONSORT diagram).

A total of 134 adults were assessed for eligibility, of whom 3 were excluded because they did not match the inclusion criteria (Figure 1). Finally, 131 volunteers were recruited and randomly distributed into four groups: the control group (*n* = 33), the WS125 group (*n* = 34), the WS250 group (*n* = 33), and the WS500 group (*n* = 31). Before the end of the 8-week intervention period, nine volunteers in the control group, seven in the WS125 group, seven in the WS250 group, and nine in the WS500 group discontinued the intervention and dropped out of the study for the reasons detailed in the study flow chart. Therefore, 99 participants completed the study and were included in the safety analyses. One subject in the WS125 group was excluded from the per-protocol analysis (efficacy). This participant, with a previous medical history of insomnia, exhibited severe levels of insomnia throughout the intervention. Therefore, analysis per protocol consisted of *n* = 24 in the control group, *n* = 26 in the WS125 group, *n* = 26 in the WS250, and *n* = 22 in the WS500 group (Figure 1)

No differences between the groups were detected regarding the number and causes of withdrawal. The compliance rate was confirmed to be very high (average of 99.5% with a range of 91.1–100%).

The demographic characteristics of the 98 subjects (70 males and 28 females) included in the per-protocol analysis are presented in Table 1. The mean age of the study population is 35 (range 20–58). The mean BMI is 24 (range 16–34). We compared age, sex, BMI, height, weight, and smoking habits among placebo and treatment groups. No significant differences between groups were found (Table 1).

### 3.2. Stress, Anxiety, and Depression Outcomes

**Figure 2:**
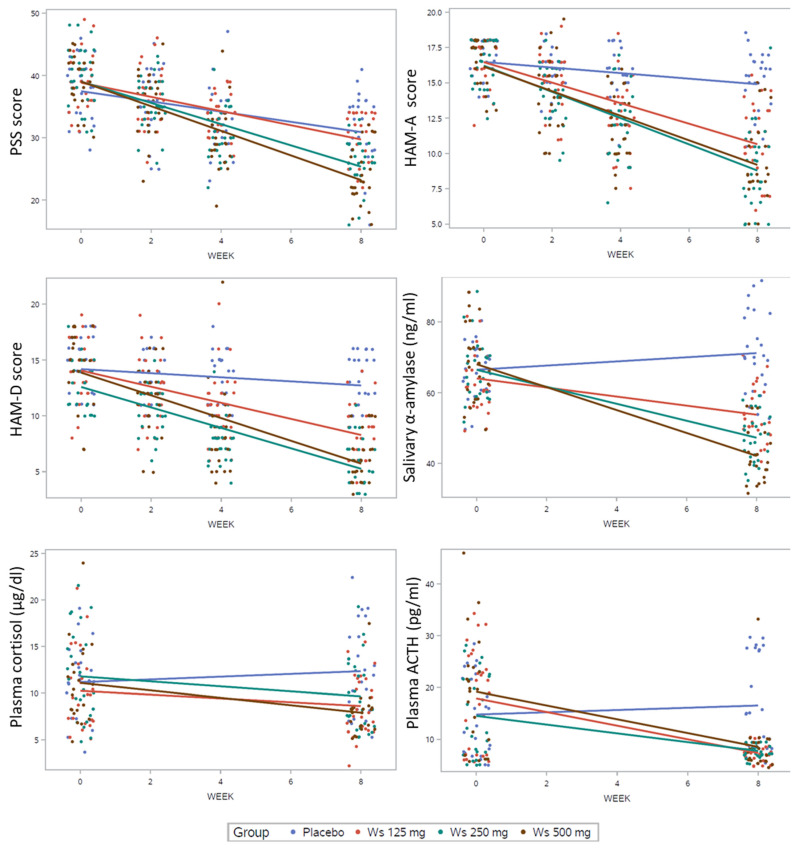

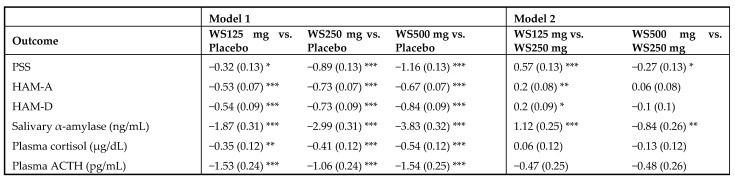
Scatter plots and slopes of PSS, HAM-A, HAM-D, and stress-related biochemical parameters among the subjects included in the per-protocol analysis consuming the placebo (blue dots and lines), WS 125 mg/d (red dots and lines), WS 250 mg/d (green dots and lines) and WS 500 mg/d (brown dots and lunes) from baseline to 8 weeks. In the table, values are the slope coefficient (SE). Model 1 compares all treatment groups versus the placebo group (reference). Model 2 compares three treatment groups, 125 mg, and 250 mg (reference). All models were adjusted for sex, age, BMI, and the corresponding baseline measure. * *p* < 0.05, ** *p* <0.01, *** *p* <0.001.

Table 2 shows the values for the Perceived Stress Scale (PSS), Hamilton’s Anxiety Scale (HAM-A), and Hamilton’s Depression Scale (HAM-D), which were evaluated during the intervention. None of these outcomes were significantly different between treatment groups at the baseline assessment (Table 2), but scores were significantly different at week 2 for HAM-A and HAM-D (*p* = 0.028 and *p* = 0.013, respectively); at week 4 for the three scores (*p* = 0.007 for PSS, *p* < 0.001 for HAM-A and *p* < 0.001 for HAM-D); and at week 8 (all three parameters with *p* < 0.001). Scatter plots and regression lines for these three outcomes and the summary statistics of the linear mixed models are presented in Figure 2. The three treatment groups (WS125 mg, WS250 mg, and WS500 mg) performed better than the placebo group, significantly decreasing the PSS, HAM-A, and HAM-D scores (Figure 2; Model 1) after 8 weeks of intervention. When comparing the different WS extract doses (Figure 2; Model 2), the effect of the extract seems to be dose-dependent in the PSS, observing a significant difference in change for PSS between the 125 mg and the 250 mg groups (*p* < 0.001) and between 500 mg and 250 mg groups (*p* < 0.05). In the case of HAM-A and HAM-D, the change from 125 mg and 250 mg was significant (*p* < 0.01 and *p* < 0.05, respectively), but it was not significant between the 250 mg and 500 mg groups.

The levels of stress-related biochemical parameters analyzed in the subjects at baseline and after 8 weeks of treatment are presented in Table 3. No significant differences at baseline were observed for any biochemical parameters. Corresponding with the observations in the stress, anxiety, and depression scores, levels of salivary amylase, plasma cortisol, and ACTH were significantly lower in all the extract groups compared to the placebo group (Tukey’s test, *p* < 0.05) after 8 weeks. No differences in DHEA-s levels and the DHEA-s-to-cortisol ratio between groups were found after 8 weeks of intervention. Scatter plots and regression lines and the statistics of the linear mixed models for salivary amylase, plasma cortisol, and plasma ACTH are shown in Figure 2. The three treatment groups (WS125 mg, WS250 mg, and WS500 mg) significantly decreased the cortisol, salivary amylase, and ACTH levels compared to the placebo after 8 weeks of intervention (Figure 2; Model 1). When comparing the different WS extract doses (Figure 2; Model 2), the effect of the extract seems to be dose-dependent in the salivary amylase—we observed a significant difference for this parameter between the 125 mg and the 250 mg groups (*p* < 0.001) and between 500 mg and 250 mg groups (*p* < 0.01). In the case of plasma cortisol and ACTH, there were no differences between the study groups.

Regarding the plasma cytokine levels analyzed during the intervention, lower levels of IL−1β, IL−6, and TNF-α were observed at the end of the study in the subjects that received the WS extracts (Tukey’s test, *p* < 0.05). No differences were observed in plasma hs-CRP levels (Table 3). Regarding the linear mixed-model results, the three treatment groups (WS125 mg, WS250 mg, and WS500 mg) exhibited significantly decreased IL−1β compared to the placebo after 8 weeks of intervention (*p* < 0.001), and we also observed a significant difference between 125 and 250 mg (Supplemental Table S1; Model 1 and Model 2). For IL−6, TNF-α, and hs-CRP levels, the 250 and 500 mg significantly reduced the cytokine levels compared to the placebo (*p* < 0.05), whereas no effect was found with the 125 mg dose (Supplemental Table S1; Model 1 and Model 2).

### 3.3. Sleep, Vitality, and Quality of Life Parameters

Table 4 presents the Pittsburgh Sleep Quality Index (PSQI), Visual Analogue Sleep Scale (VAS-S), and Visual Analogue Energy, Vitality and Drive Scale (VAS-E). No parameters significantly differ at baseline among the placebo group and treatment groups, and subjects taking the WS extracts show improvement during the intervention compared to the placebo. After 2 weeks of treatment, VAS-E scores were significantly different between groups (*p* = 0.029); at week 4 significant differences between groups in VAS-S and VAS-E were observed (*p* < 0.001), whereas, at week 8, all scores in the three WS extract groups presented improvement compared to the placebo (Tukey’s test, *p* < 0.05). Results of the linear mixed models (Supplemental Table S1, Model 2) show that the three treatment groups (WS125 mg, WS250 mg, and WS500 mg) improve the VAS-S and VAS-E compared to the placebo (*p* < 0.001), whereas the PSQI shows significant improvement in the subjects taking the 250 mg (*p* < 0.01) and 500 mg (*p* < 0.001) doses vs. placebo. There were no significant differences between the three different WS extract doses except for the VAS-E between the WS125 mg and WS250 mg groups (*p* < 0.001; Supplemental Table S1, Model 2).

The four domains of the WHO quality of life scale: physical health, psychological health, social relationships, and environmental health are presented in Supplemental Table S2. No quality-of-life measures significantly differed at baseline among the placebo group and treatment groups. During the intervention, domain 1 (physical health) presented significant differences between groups at week 8 (*p* = 0.0045), and domain 2 (psychological health) presented significant differences between groups at week 4 (*p* = 0.0013).

### 3.4. Safety Parameters and Adverse Events

Safety parameters were analyzed in all subjects that completed the intervention (*n* = 99). Pre- and post-intervention safety measures comprised a full blood count (Supplemental Table S3), glucose levels, lipid profile, and thyroid, liver, and renal function parameters (Table 5). No significant changes between the baseline and after 8 weeks of intervention were found except in the albumin/globulin ratio levels in the WS 250 group (*p* < 0.05; Table 5), in the ESR first hour, and the total platelet count in the WS500 mg group (*p* < 0.05; Supplemental Table S1). However, all parameters were within reference ranges, and the observed changes could be considered normal life changes not related to the intervention.

No serious adverse events were reported during the intervention. Adverse events were reported in 48% of study participants, as measured by the Systematic Assessment for Treatment Emergent Effects (SAFTEE) during the 8 weeks of intervention. The majority of adverse events were heartburn, abdominal discomfort, and trouble sleeping, along with other minor issues. There were no obvious trends in reported adverse events observed between the treatment groups and the placebo. Moreover, the observations revealed that 1 week after the end of the intervention (9-week follow-up), the withdrawal effects of WS extract were minimal or nonexistent. Based on these observations, it is suggested that the general safety of the WS extract is supported.

## 4. Discussion

The present study shows that the consumption of the standardized *Withania somnifera* leaves and root extract (Sensoril^®^) at three different doses (125 mg, 250 mg, and 500 mg) safely and effectively reduces stress, anxiety, and depression in chronically stressed subjects after an 8-week intervention. Specifically, this WS extract dose-dependently and significantly attenuated stress, as assessed by the Perceived Stress Scale (PSS)—a validated and robust tool for assessing the appraisal of stress [17], and reduced levels of well-known biomarkers related to stress, such as plasma cortisol, ACTH, and salivary α-amylase [3]. Moreover, the extract also improved sleep, vitality, and quality-of-life parameters in these subjects.

Consistent with our results, there are several randomized clinical trials that reported WS extract to have a mood-enhancing effect in stressed adults [18]. However, to our knowledge, this is the first study that observed a dose-dependent effect using three different doses of fully elucidated aqueous extract for stress amelioration measured by both questionnaires and biomarkers; and reported a positive effect in the lowest dose tested so far for stress (125 mg/day) after 8 weeks of intervention. The 14-item PSS is one of the most widely used tools to measure psychological stress, and reliability and validity have been broadly demonstrated [19,20]. Although the PSS is not a clinical diagnostic instrument, it is considered a valid tool to measure perceived helplessness and self-efficacy—two factors known to be distinguishing features of subjectively perceived stress according to the transactional stress model (20). Moreover, the results observed in the PSS were accompanied by a significant reduction in both the HAM-A and HAM-D scores [21,22], showing the effectiveness of this WS extract in ameliorating anxiety and depression symptoms related to stress.

The biological markers measured to address the mechanisms of action by which WS extract might exert its anti-stress effect included several hormones related to the HPA axis activity, and to the autonomic nervous system (ANS); and several pro-inflammatory cytokines, which are related to innate immunity [23]. The activation of the HPA axis is a fundamental trigger of the stress response [24]. Corticotrophin-releasing hormone (CRH) plays a key role in the activation of the HPA axis with the release of glucocorticoids (cortisol), which may act on many organ systems to redirect sources of energy to accommodate actual or projected demand [24]. The WS extract may have an attenuating effect on HPA-axis activity, as we observed decreasing levels of blood cortisol and ACTH after 8 weeks of intervention. Interestingly, in this study, we observed a dose-dependent significantly lowered concentration of salivary α-amylase, based on our observations of the PSS scores of these stress volunteers. Since α-amylase is produced in acinar cells, which are innervated by sympathetic and parasympathetic branches of the ANS, salivary α-amylase concentration has been used as a reliable and sensitive stress marker [25]. Finally, some pro-inflammatory cytokines such as interleukin (IL)−6, IL−1β, and tumor necrosis factor (TNF)-α, which are involved in innate immunity, have been suggested to respond to acute and chronic psychosocial stress [26]. Therefore, the decreased levels of blood IL−1β, IL−6, and TNF-α that we observed in these volunteers after 8 weeks of intervention suggest that another potential mechanism of the ashwagandha’s stress-amelioration effect may be via its antioxidant and anti-inflammatory effects [27].

Moreover, we observed positive effects on the sleep and vitality parameters of these subjects. Although the relationship between chronic stress and sleep alteration is well known [28], few clinical studies controlled both stress and sleep parameters in the same intervention [29]. The HPA axis plays an important role in alterations of the sleep–wake cycle, secondary to exposure to chronic stressors [30]. Therefore, the improvement in the sleep quality reported in the volunteers consuming the WS extract may be related to the attenuation of HPA-axis activity. However, further studies should be conducted to demonstrate the efficacy of WS extract in sleep regulation.

Another aim of this study was to support the safety profile of ashwagandha. We demonstrated that consumption of this WS extract for 8 weeks at 125 mg, 250 mg, and 500 mg per day is safe and well-tolerated. No adverse events related to Sensoril consumption were recalled. Together with the fact that we found no changes in triiodothyronine 3 (T3), thyroxine (T4), thyroid-stimulating hormone (TSH), creatinine, glucose, proteins, albumin, cholesterol, triglycerides, bilirubin, and urea levels, we can confirm the safety of the Sensoril WS extract.

A limitation of this study was that the recruitment and follow-up of the intervention were disturbed by the COVID-19 pandemic, resulting in a high dropout rate due to not attending follow-up visits. Despite every effort to replace dropouts, the COVID-19 situation finally led to a longer length duration of the study (September 2019 to October 2022) and to a lower-than-expected final sample size. Nevertheless, the study was able to show significant differences between study groups for several parameters.

## 5. Conclusions

The consumption of the standardized *Withania somnifera* extract of leaves and roots (Sensoril^®^) at doses of 125 mg, 250 mg, and 500 mg for 8 weeks safely and effectively reduces stress parameters in chronically stressed subjects. These results were consistently observed in both validated questionnaires and using stress-related biomarkers in a dose-dependent manner, confirming the lowest dose for stress management (125 mg per day) using a standardized aqueous WS extract of roots and leaves. Moreover, positive effects on the sleep and vitality parameters of these subjects were observed. All of this, together with the fact that the study subjects did not suffer from adverse effects and that the WS extract was well-tolerated, supports the role of Sensoril^®^ as a safe and effective strategy for mild-to-moderate stress amelioration.

## Figures and Tables

**Table 1:** Demographic characteristics of study participants included in the per-protocol analysis (*n* = 98).

	Placebo (*n* = 24)	WS125 mg (*n* = 26)	WS250 mg (*n* = 26)	WS500 mg (*n* = 22)	*p* between Groups
Age (years)	35.58 ± 9.80	35.62 ± 9.25	35.38 ± 9.13	34.55 ± 11.07	0.9807
Sex					
Females (*n*, %)	7(29.17%)	11(42.31%)	6(23.08%)	4(18.18%)	0.2662
Males (*n*, %)	17(70.83%)	15(57.69%)	20(76.92%)	18(81.82%)	
BMI (kg/m^2^)	24.26 ± 3.43	23.81 ± 3.91	23.21 ± 3.76	23.63 ± 3.51	0.7911
Weight(kg)	63.80 ± 8.41	60.10 ± 10.56	60.14 ± 12.35	62.66 ± 9.02	0.4949
Height(cm)	162.55 ± 10.86	158.91 ± 9.12	160.70 ± 10.35	163.09 ± 7.96	0.4240
Smoking (yes)	3(12.50%)	2(7.69%)	2(7.69%)	2(9.09%)	0.9286

Values are mean ± SD for continuous variables and *n* (%) for categorical variables. *p* indicates differences between the placebo and the treatment groups (ANOVA test).

**Table 2:** Stress, anxiety, and depression scores of the subjects consuming the placebo or the WS extracts (125, 250, and 500 mg) at baseline, 2 weeks, 4 weeks, and 8 weeks (per protocol, *n* = 98).

	Placebo (*n* = 24)	WS125 mg (*n* = 26)	WS250 mg (*n* = 26)	WS500 mg (*n* = 22)	*p* between Groups
Perceived Stress Scale (PSS)					
Baseline	37.96 ± 4.99	39.15 ± 4.42	39.92 ± 4.85	39.36 ± 4.25	0.507
2 weeks	36.00 ± 5.21	36.54 ± 4.76	35.54 ± 4.31	35.36 ± 4.96	0.828
4 weeks	32.92 ± 5.04 ^a^	33.65 ± 3.30 ^a^	30.38 ± 4.23 ^ab^	29.91 ± 5.14 ^ab^	0.007
8 weeks	31.50 ± 5.76 ^a^	30.04 ± 4.06 ^a^	26.27 ± 3.98 ^b^	23.73 ± 4.67 ^b^	<0.001
Hamilton’s Anxiety Scale (HAM-A)					
Baseline	16.90 ± 1.33	16.67 ± 1.64	16.48 ± 1.69	16.48 ± 1.64	0.770
2 weeks	15.73 ± 1.57	15.44 ± 2.00	14.21 ± 2.01	14.36 ± 2.82	0.028
4 weeks	15.38 ± 1.97 ^a^	12.50 ± 2.45 ^b^	12.19 ± 2.50 ^b^	12.07 ± 2.39 ^b^	<0.001
8 weeks	15.15 ± 2.10 ^a^	11.08 ± 2.53 ^b^	8.98 ± 2.91 ^c^	9.50 ± 2.89 ^c^	<0.001
Hamilton’s Depression Scale (HAM-D)					
Baseline	14.83 ± 2.33	14.54 ± 2.85	13.19 ± 2.25	14.43 ± 3.22	0.139
2 weeks	13.17 ± 2.08 ^a^	12.23 ± 3.01 ^ab^	10.69 ± 2.24 ^b^	11.82 ± 3.08 ^ab^	0.013
4 weeks	13.15 ± 2.41 ^a^	10.77 ± 3.24 ^b^	7.81 ± 1.98 ^c^	8.70 ± 3.92 ^bc^	<0.001
8 weeks	13.04 ± 2.42 ^a^	8.58 ± 2.52 ^b^	5.83 ± 2.12 ^c^	6.27 ± 2.27 ^c^	<0.001

Values are mean ± SD. *p* indicates differences between groups at each time point. Different letters mean significant differences between groups (ANOVA followed by Tukey’s test).

**Table 3:** Stress-related biochemical parameters of the subjects consuming the placebo or the WS extracts (125, 250, and 500 mg) at baseline and 8 weeks (per protocol, *n* = 98).

	Placebo (*n* = 24)	WS125 mg (*n* = 26)	WS250 mg (*n* = 26)	WS500 mg (*n* = 22)	*p* between Groups
Salivary α-amylase (ng/mL)					
Baseline	66.52 ± 7.68	64.05 ± 9.53	66.49 ± 8.58	68.07 ± 10.78	0.498
8 weeks	71.22 ± 11.44 ^a^	53.79 ± 7.62 ^b^	47.27 ± 6.78 ^c^	42.14 ± 7.47 ^c^	<0.001
Plasma cortisol (μg/dL)					
Baseline	11.16 ± 3.84	10.23 ± 4.20	11.78 ± 4.74	11.08 ± 4.24	0.632
8 weeks	12.34 ± 4.73 ^a^	8.61 ± 3.32 ^b^	9.65 ± 3.77 ^ab^	7.90 ± 2.51 ^b^	<0.001
Plasma DHEA-S (μg/dL)					
Baseline	181.78 ± 81.22	180.61 ± 92.76	211.96 ± 139.12	227.44 ± 123.58	0.389
8 weeks	176.13 ± 85.45	170.72 ± 87.15	198.16 ± 134.65	199.22 ± 106.28	0.696
DHEAS-to-Cortisol ratio					
Baseline	19.01 ± 14.14	19.90 ± 12.02	19.06 ± 12.44	22.21 ± 12.72	0.816
8 weeks	16.50 ± 11.13	21.46 ± 10.90	23.09 ± 17.93	25.38 ± 12.85	0.153
Plasma ACTH (pg/mL)					
Baseline	14.77 ± 7.68	17.88 ± 9.66	14.52 ± 8.11	19.17 ± 10.69	0.210
8 weeks	16.52 ± 9.05 ^a^	7.37 ± 1.76 ^b^	7.77 ± 1.24 ^b^	8.55 ± 5.81 ^b^	<0.001
Plasma hs-CRP (mg/L)					
Baseline	0.83 ± 0.37	0.82 ± 0.39	0.82 ± 0.27	0.97 ± 0.56	0.504
8 weeks	1.34 ± 2.12	0.76 ± 0.36	0.67 ± 0.20	0.75 ± 0.36	0.124
Plasma IL−1β (pg/mL)					
Baseline	21.78 ± 13.09	25.06 ±10.21	26.23 ±15.58	25.94 ± 14.02	0.639
8 weeks	19.68 ± 11.67 ^a^	17.00 ± 7.27 ^ab^	13.47 ± 6.06 ^b^	11.59 ± 5.34 ^b^	0.004
Plasma IL−6 (pg/mL)					
Baseline	276.46 ± 135.26	261.77 ± 136.33	258.52 ± 117.47	266.23 ± 114.06	0.963
8 weeks	249.75 ± 118.29 ^a^	187.56 ± 88.29 ^ab^	177.96 ± 78.21 ^b^	154.36 ± 69.58 ^b^	0.004
Plasma TNF-α (pg/mL)					
Baseline	1070.8 ± 415.5	1086.9 ± 234.0	1199.4 ± 645.9	1130.0 ± 419.4	0.749
8 weeks	1067.29 ± 348.6 ^a^	886.5 ± 259.9 ^ab^	792.0 ± 365.4 ^b^	788.2 ± 390.1 ^b^	0.018

Values are mean ± SD. *p* indicates differences between groups at each time point. Different letters mean significant differences between groups (ANOVA followed by Tukey’s test).

**Table 4:** Sleep and vitality scores of the subjects consuming the placebo or the WS extracts (125, 250, and 500 mg) at baseline, 2 weeks, 4 weeks, and 8 weeks (per protocol, *n* = 98).

	Placebo (*n* = 24)	WS125 mg (*n* = 26)	WS250 mg (*n* = 26)	WS500 mg (*n* = 22)	*p* between Groups
Pittsburgh Sleep Quality Index (PSQI).					
Baseline	16.17 ± 5.37	15.31 ± 7.04	14.08 ± 5.49	18.41 ± 7.39	0.128
2 weeks	12.58 ± 4.95	11.81 ± 5.62	10.31 ± 4.54	12.64 ± 6.76	0.408
4 weeks	11.04 ± 4.69	10.58 ± 5.89	7.31 ± 4.70	9.23 ± 5.49	0.055
8 weeks	10.96 ± 4.41 ^a^	7.58 ± 2.97 ^b^	5.00 ± 2.65 ^c^	7.45 ± 3.86 ^bc^	<0.001
Visual Analogue Sleep Scale (VAS-S).					
Baseline	4.88 ± 1.40	5.08 ± 1.60	5.56 ± 1.56	4.52 ± 1.64	0.140
2 weeks	5.25 ± 1.29	5.77 ± 1.73	6.35 ± 1.23	5.84 ± 1.51	0.075
4 weeks	4.67 ± 1.09 ^a^	6.08 ± 1.72 ^b^	7.13 ± 1.49 ^b^	6.50 ± 1.74 ^b^	<0.001
8 weeks	4.63 ± 1.13 ^a^	7.27 ± 1.40 ^b^	8.31 ± 1.23 ^c^	7.50 ± 1.60 ^bc^	<0.001
Visual Analogue Energy, Vitality and Drive Scale (VAS-E).					
Baseline	4.27 ± 1.80	4.81 ± 1.96	4.63 ± 1.71	4.82 ± 1.26	0.661
2 weeks	4.69 ± 1.41 ^a^	5.58 ± 1.65 ^ab^	5.83 ± 1.33 ^b^	5.82 ± 1.56 ^ab^	0.029
4 weeks	4.67 ± 1.17 ^a^	5.73 ± 1.69 ^ab^	6.88 ± 1.68 ^c^	6.77 ± 1.58 ^bc^	<0.001
8 weeks	4.73 ± 1.19 ^a^	6.67 ± 1.43 ^b^	7.87 ± 1.62 ^c^	7.89 ± 1.53 ^c^	<0.001

Values are mean ± SD. *p* indicates differences between groups at each time point. Different letters mean significant differences between groups (ANOVA followed by Tukey’s test).

**Table 5:** Safety parameters of the subjects consuming the placebo or the WS extracts (125, 250, and 500 mg) at baseline and after 8 weeks of intervention (safety population, *n* = 99).

Title	Week	Placebo (*n* = 24)	WS125 mg (*n* = 27)	WS250 mg (*n* = 26)	WS500 mg (*n* = 22)
Glucose (mg/dL)	0	87.04 ± 6.69	88.46 ± 6.27	92.69 ± 13.64	95.66 ± 30.71
	8	87.68 ± 6.35	88.12 ± 7.21	90.65 ± 10.23	95.73 ± 23.79
Total Cholesterol (mg/dL)	0	179.92 ± 47.85	175.19 ± 30.74	176.96 ± 37.87	180.91 ± 36.02
	8	174.88 ± 42.53	168.11 ± 39.51	170.96 ± 35.12	178.95 ± 34.66
HDL Cholesterol (mg/dL)	0	41.83 ± 8.98	44.07 ± 8.49	47.19 ± 11.77	43.00 ± 11.69
	8	43.75 ± 11.47	43.07 ± 10.02	44.12 ± 8.00	41.09 ± 6.10
LDL Cholesterol (mg/dL)	0	114.67 ± 39.61	108.85 ± 24.98	105.96 ± 28.35	113.64 ± 29.49
	8	106.50 ± 29.58	103.59 ± 32.24	103.69 ± 28.48	112.91 ± 30.43
VLDL Cholesterol (mg/dL)	0	23.42 ± 8.68	22.26 ± 8.10	23.81 ± 9.65	24.27 ± 8.01
	8	24.63 ± 9.50	21.44 ± 7.42	23.15 ± 9.43	24.95 ± 8.28
Triglycerides (mg/dL)	0	131.21 ± 79.21	123.44 ± 67.65	128.46 ± 72.53	129.41 ± 52.89
	8	122.96 ± 48.71	118.85 ± 81.48	134.38 ± 85.83	139.18 ± 68.72
Cholesterol/HD	0	4.35 ± 0.97	4.05 ± 0.70	3.90 ± 1.06	4.36 ± 0.90
	8	4.10 ± 0.85	3.97 ± 0.86	3.91 ± 0.83	4.40 ± 0.82
LDL/HDL	0	2.78 ± 0.88	2.53 ± 0.64	2.36 ± 0.83	2.77 ± 0.80
	8	2.50 ± 0.68	2.45 ± 0.74	2.40 ± 0.68	2.78 ± 0.73
T3 (ng/mL)	0	1.12 ± 0.22	1.17 ± 0.21	1.18 ± 0.17	1.19 ± 0.19
	8	1.17 ± 0.19	1.21 ± 0.22	1.15 ± 0.21	1.24 ± 0.22
T4 (μg/dL)	0	7.67 ± 1.92	8.00 ± 1.87	8.18 ± 1.31	8.36 ± 1.41
	8	7.74 ± 1.49	8.61 ± 1.44	8.21 ± 1.54	8.37 ± 1.47
TSH (μIU/mL)	0	2.85 ± 1.34	3.21 ± 2.38	3.34 ± 2.96	2.76 ± 2.27
	8	2.89 ± 1.95	3.20 ± 3.66	3.04 ± 2.19	3.07 ± 2.86
Total Protein (g/dL)	0	7.48 ± 0.43	7.30 ± 0.36	7.40 ± 0.43	7.52 ± 0.36
	8	7.50 ± 0.45	7.27 ± 0.41	7.42 ± 0.40	7.55 ± 0.42
Albumin (g/dL)	0	4.28 ± 0.28	4.32 ± 0.36	4.44 ± 0.29	4.36 ± 0.27
	8	4.25 ± 0.31	4.29 ± 0.27	4.38 ± 0.27	4.43 ± 0.25
Globulin (g/dL)	0	3.20 ± 0.33	2.98 ± 0.33	2.96 ± 0.36	3.15 ± 0.40
	8	3.23 ± 0.34	2.99 ± 0.29	3.03 ± 0.29	3.12 ± 0.46
Albumin/Globulin	0	1.35 ± 0.16	1.48 ± 0.26	1.52 ± 0.22	1.41 ± 0.23
	8	1.34 ± 0.15	1.45 ± 0.16	1.46 ± 0.16 *	1.46 ± 0.28
SGOT (U/L)	0	29.91 ± 12.65	25.50 ± 5.82	30.31 ± 10.44	37.74 ± 28.81
	8	29.37 ± 10.12	27.57 ± 14.13	30.60 ± 17.19	30.87 ± 15.83
SGPT (U/L)	0	34.51 ± 23.93	25.20 ± 9.85	33.10 ± 21.95	42.31 ± 33.19
	8	33.33 ± 19.16	28.63 ± 16.53	38.60 ± 45.18	32.63 ± 17.18
ALP (u/L)	0	85.99 ± 17.64	73.60 ± 15.43	74.07 ± 17.47	83.85 ± 18.22
	8	90.22 ± 43.46	72.11 ± 16.08	73.70 ± 15.28	84.98 ± 23.55
Total_Bilirubin(mg/dL)	0	0.77 ± 0.34	0.87 ± 0.49	0.84 ± 0.39	0.80 ± 0.29
	8	0.79 ± 0.35	0.82 ± 0.36	0.78 ± 0.25	0.74 ± 0.23
Conjugated Bilirubin (mg/dL)	0	0.21 ± 0.04	0.22 ± 0.05	0.21 ± 0.03	0.20 ± 0.04
	8	0.22 ± 0.04	0.22 ± 0.05	0.21 ± 0.03	0.20 ± 0.02
Unconjugated Bilirubin (mg/dL)	0	0.56 ± 0.30	0.65 ± 0.45	0.63 ± 0.36	0.60 ± 0.26
	8	0.58 ± 0.32	0.60 ± 0.31	0.57 ± 0.24	0.53 ± 0.22
Urea (mg/dL)	0	20.52 ± 4.15	21.34 ± 4.50	20.71 ± 4.09	20.46 ± 3.32
	8	20.54 ± 3.03	21.07 ± 4.78	21.85 ± 4.83	20.29 ± 3.58
Creatinine (mg/dL)	0	0.92 ± 0.18	0.90 ± 0.16	0.90 ± 0.14	0.93 ± 0.13
	8	0.89 ± 0.14	0.91 ± 0.14	0.91 ± 0.16	0.93 ± 0.10

Values are mean ± SD. Asterisk (*) indicates *p* < 0.05 between baseline and the end of intervention (8 weeks) within groups (*t*-test analysis).

## Data Availability

The original contributions presented in the study are included in the article/Supplementary Material, further inquiries can be directed to the corresponding author.

## Supplementary Materials

The following supporting information can be downloaded at: https://www.mdpi.com/article/10.3390/nu16091293/s1, Table S1: Linear Mixed Models (difference-in-difference evaluation) of all parameters evaluated in the subjects consuming the placebo or the WS extracts (125, 250, and 500 mg) during the 8 weeks of intervention (per-protocol analysis, *n* = 98); Table S2: Quality of life outcomes at each study time point among treatment and placebo groups (per-protocol analysis, *n* = 98); Table S3: Complete hemogram evaluating the subjects consuming the placebo or the WS extracts (125, 250, and 500 mg) at baseline and after the 8 weeks of intervention (safety population, *n* = 99).
