# Supplementary material for: Effects of Withania somnifera Extract in Chronically Stressed Adults: A Randomized Controlled Trial
Source: Nutrients. 2024 Apr 26;16(9):1293. doi: 10.3390/nu16091293 (PMC11085552; doi:10.3390/nu16091293)
Supplement: Supplementary file 1 [file nutrients-16-01293-s001.zip › nutrients-2956892-supplementary.pdf]

Supplementary Materials

Supplement Table S1. Linear Mixed Models (difference-in-difference evaluation) of all parameters evaluated in the subjects consuming the placebo or the WS extracts (125, 250 and 500 mg) during the 8-weeks of intervention. (per-protocol analysis, n=98)

	**Model 1**			**Model 2**	
**Outcome**	**WS125 mg vs Placebo**	**WS250 mg vs Placebo**	**WS500 mg vs Placebo**	**WS125 mg vs WS250 mg**	**WS500 mg vs WS250 mg**
**Plasma DHEA-S (μg/dl)**	-0.53 (1.7)	-1.02 (1.7)	-2.82 (1.77)	0.49 (1.79)	-1.8 (1.87)
**DHEAS to Cortisol ratio**	0.51 (0.3)	0.82 (0.3)**	0.71 (0.31)*	-0.31 (0.33)	-0.11 (0.34)
**Plasma hs-CRP (mg/l)**	-0.07 (0.04)	-0.08 (0.04)*	-0.09 (0.04)*	0.01 (0.01)	-0.01 (0.01)
**Plasma IL-1β (pg/ml)**	-74.37 (21.64)***	-133.12 (21.64)***	-153.13 (22.56)***	58.75 (22.05)**	-20.01 (23.03)
**Plasma IL-6 (pg/ml)**	-593.79 (314.07)	-673.12 (314.07)*	-1064.44 (327.49)**	79.33 (278.55)	-391.32 (290.94)
**Plasma TNF-α (pg/ml)**	-2460.54 (1341.78)	-5048.04 (1341.78)***	-4228.46 (1399.11)**	2587.5 (1379.49)	819.58 (1440.83)
**PSQI**	-0.32 (0.17)	-0.51 (0.17)**	-0.7 (0.17)***	0.19 (0.17)	-0.19 (0.17)
**VAS-S**	0.32 (0.04)***	0.39 (0.04)***	0.41 (0.05)***	-0.08 (0.05)	0.01 (0.05)
**VAS-E**	0.17 (0.05)***	0.35 (0.05)***	0.33 (0.05)***	-0.18 (0.05)***	-0.02 (0.05)
**WHOQOL1**	-0.01 (0.09)	0.23 (0.09)*	0.21 (0.09)*	-0.24 (0.08)**	-0.02 (0.09)
**WHOQOL2**	-0.14 (0.07)*	0.09 (0.07)	0.01 (0.07)	-0.23 (0.07)**	-0.07 (0.07)
**WHOQOL3**	-0.05 (0.04)	-0.01 (0.04)	-0.03 (0.04)	-0.04 (0.04)	-0.02 (0.04)
**WHOQOL4**	-0.03 (0.11)	0.06 (0.11)	-0.03 (0.11)	-0.09 (0.1)	-0.09 (0.11)

Values are the slope coefficient (SE). Model 1 compares all treatment groups versus the placebo group (reference) and. Model 2 compares three treatment groups, 125mg, 250mg (reference). All models were adjusted for sex, age, BMI, and the corresponding baseline measure. *p<0.05, ** p <0.01, *** p <0.001.

Supplemental Table S2. Quality of life outcomes at each study timepoint among treatment and placebo groups (per-protocol analysis, n=98)

	**Placebo**	**WS125 mg**	**WS250mg**	**WS500mg**	**P-value**
Domain 1 (Physical health). mean ± SD					
Baseline	16.08±2.28	16.88±2.92	16.50±2.98	16.32±3.21	0.7905
2 weeks	17.04±2.07	17.96±2.46	18.27±2.59	17.41±2.89	0.3185
4 weeks	18.71±2.84	18.58±2.18	19.77±2.72	18.68±3.14	0.3595
8 weeks	19.25±2.83^{a}	20.12±2.70^{ab}	21.62±1.68^{b}	21.18±2.46^{b}	0.0045
Domain 2 (Psychological health). mean ± SD					
Baseline	16.21±2.38	16.58±2.53	16.35±2.77	16.95±2.61	0.7761
2 weeks	16.29±2.33	16.23±2.07	16.92±2.21	16.95±2.46	0.5325
4 weeks	17.46±2.19^{ab}	15.92±2.43^{b}	18.35±1.72^{a}	18.18±2.95^{a}	0.0013
8 weeks	17.46±2.19	16.81±2.12	18.35±1.72	18.27±3.01	0.0575
Domain 3 (Social relationship). mean ± SD					
Baseline	7.75±2.09	8.46±2.02	8.38±1.83	8.27±1.49	0.5470
2 weeks	7.83±2.06	8.27±1.95	8.38±1.75	8.55±1.26	0.5658
4 weeks	8.17±1.97	8.88±1.93	8.58±1.81	8.73±1.45	0.5495

8 weeks	8.63±1.79	8.88±1.77	9.19±1.63	8.95±1.50	0.6960
Domain 4(Environmental health). mean ± SD					
Baseline	21.25±3.31	21.54±3.66	21.85±3.98	22.36±4.44	0.7880
2 weeks	21.13±3.55	22.00±3.43	22.81±4.00	23.00±4.01	0.2992
4 weeks	19.04±3.53	19.65±3.38	21.04±3.28	20.91±4.06	0.1484
8 weeks	23.83±3.43	24.04±3.50	25.08±3.47	24.86±3.60	0.5263

Values are mean ± SD. *P* indicates differences between groups at each time point. Different letters mean significant differences between groups (ANOVA followed by Tukey's test)

Supplemental Table S3. Complete hemogram evaluated in the subjects consuming the placebo or the WS extracts (125, 250 and 500 mg) at baseline at after the 8-weeks of intervention (safety population, n=99)

	Week	Placebo	Ws 125 mg	Ws 250 mg	Ws 500 mg
Hemoglobin (g/dL)	0	13.43±1.64	13.21±1.93	13.69±1.74	14.00±1.90
	8	13.48±1.58	13.13±1.50	13.77±1.63	13.90±2.05
Total RBC (millions/cumm)	0	4.70±0.59	4.69±0.56	4.75±0.56	4.93±0.61
	8	4.65±0.57	4.58±0.54	4.79±0.57	4.91±0.70
Total WBC (per cumm)	0	6708.33±1845.07	6455.56±1831.63	6530.77±1565.83	6613.64±1357.62
	8	7029.17±2472.01	6140.74±1635.02	6819.23±1985.55	6581.82±1822.51
Total Platelet (per cumm)	0	283250.00±42408.21	266037.04±43318.57	263884.62±44003.02	286090.91±29258.96
	8	268916.67±48388.75	265222.22±36808.58	265230.77±46791.72	269000.00±34097.90*
Neutrophill (%)	0	63.21±6.46	65.48±7.75	63.58±6.07	62.41±6.68
	8	61.92±10.49	65.41±6.11	63.50±10.50	63.91±4.97
Lymphocyte (%)	0	30.13±5.30	28.44±7.73	30.12±6.00	31.00±6.21
	8	31.38±9.68	27.63±5.69	28.42±6.07	29.05±5.67
Monocyte (%)	0	2.00±0.00	2.00±0.00	1.96±0.20	2.00±0.00
	8	2.00±0.00	1.96±0.19	2.00±0.00	2.00±0.00
Eosinophill (%)	0	4.67±2.08	4.07±1.96	4.35±2.23	4.59±2.79
	8	4.46±3.04	4.85±2.55	6.19±6.78	5.05±3.66
PCV (%)	0	40.58±4.94	39.86±5.53	41.37±5.22	42.32±5.68
	8	40.88±4.76	39.89±4.21	41.79±4.96	42.32±6.25
MCV (fL)	0	86.46±1.13	84.98±4.48	86.97±2.25	85.79±2.06
	8	88.47±5.32	87.47±5.48	87.17±3.39	86.48±2.77
MCH (pg)	0	28.61±0.36	28.13±1.68	28.77±0.84	28.37±0.69
	8	29.17±1.83	28.77±2.28	28.68±1.28	28.42±1.05
MCHC (g/dL)	0	33.09±0.10	33.10±0.37	33.09±0.27	33.07±0.11
	8	32.99±0.32	32.83±1.01	32.90±0.51	32.86±0.55
ESR_1st hour (mm/h)	0	18.21±10.09	16.19±10.54	14.88±10.09	10.64±8.95
	8	16.54±9.35	15.56±9.84	14.08±10.40	14.73±12.18*

Values are mean ± SD. Asterisk (*) indicates p<0.05 between baseline and the end of intervention (8 weeks) within groups
